# End-stage renal disease reduces the expression of drug-metabolizing cytochrome P450s

**DOI:** 10.1007/s43440-020-00127-w

**Published:** 2020-07-08

**Authors:** Máté Tamás Déri, Ádám Ferenc Kiss, Katalin Tóth, József Paulik, Enikő Sárváry, László Kóbori, Katalin Monostory

**Affiliations:** 1 Institute of Enzymology, Research Centre for Natural Sciences, Magyar tudósok 2, 1117 Budapest, Hungary; 2 Nucleotest Bio Ltd., Tündérliget 3/2, 1038 Budapest, Hungary; 3 Department of Transplantation and Surgery, Semmelweis University, Baross 23, 1082 Budapest, Hungary

**Keywords:** End-stage renal disease, Cytochrome P450, RNA extraction, Reverse transcription, Duplex quantitative PCR

## Abstract

**Background:**

End-stage renal disease is an irreversible status of kidney dysfunction that reduces both renal and non-renal drug clearance. Accumulation of uremic toxins seems to modify the activities of drug-metabolizing cytochrome P450 (CYP) enzymes. The aim of the present work was to refine gene expression analysis for efficient and accurate quantification of CYP mRNAs in patients’ leukocytes.

**Methods:**

We compared six liquid–liquid extraction reagents for RNA isolation and five reverse transcriptase kits for RNA-to-cDNA conversion, and developed quantitative polymerase chain reaction methods for duplex measurements of CYP target genes and the reference gene. The expression of CYP1A2, CYP2C9, CYP2C19 and CYP3A4 in patients with end-stage kidney disease (*N* = 105) and in organ donors with healthy kidney function (*N* = 110) was compared.

**Results:**

Regarding the RNA yield and purity, TRIzol, Trizolate and TRI reagents were equal; however, TRI reagent was the most advantageous in terms of financial cost. Reverse transcription using Maxima First Strand cDNA Synthesis kit appeared to be the most efficient with the widest range for quantification of the target transcript. The refined method with the detection of various CYPs and the reference gene in duplex PCR efficiently quantified even the low-level CYP expression. In leukocytes of patients with end-stage renal disease, all four CYPs were expressed at significantly lower level than in organ donors with normal kidney function (*p* < 0.0001).

**Conclusions:**

Reduced CYP expression was a direct evidence of transcriptional down-regulation of CYP genes in patients with impaired kidney function.

## Introduction

The incidence of chronic kidney disease is 10–16% in European adult population, and approximately 0.1–0.2% of these patients suffers from end-stage renal disease [1, 2]. The major risk factors are diabetes mellitus, hypertension, obesity and older age. Chronic kidney disease is primarily associated with the progression to kidney failure and with higher rates of cardiovascular disease (e.g., dysrhythmias, heart failure and stroke) [3, 4]; while, further serious complications include anemia, bone disease and decreased immune response [5]. End-stage renal disease is an irreversible status, accompanied with proteinuria and substantial reduction of glomerular filtration rate; therefore, patients with kidney failure require renal replacement therapy, including dialysis or kidney transplantation, unless fatal outcome can be predicted [1, 2, 6]. Although kidney transplantation is the optimal way of renal replacement therapy, the majority of patients receive dialysis because of the shortage of transplantable organs or contraindication to kidney transplantation [1]. The chronic dialysis improves life expectancies of patients; however, the inter-dialytic fluid accumulation and the fluctuations in electrolytes and uremic toxins may cause additional complications, such as blood pressure fluctuation, myocardial hypoperfusion, reduction in cerebral perfusion and neurological complications [7–9].

Because of the comorbid conditions, the medication of patients with chronic kidney disease is complex with the highest pill burden amongst any chronic disease patients. The progressive decline in kidney function reduces the renal drug clearance, requiring dose adjustment. However, the non-renal drug metabolism (hepatic and intestinal) has also been demonstrated to decrease in patients with impaired kidney function, resulting in clinically significant changes in drug exposure [10, 11]. Cytochrome P450 (CYP) enzymes belonging to the CYP1-3 enzyme families play a dominant role in drug metabolism [12]. CYP activities can display more than 100-fold inter-individual variability [13], which is partly explained by genetic polymorphism. Transcriptional induction and suppression of CYP genes or inhibition of CYP enzyme activities can result in substantial modification in drug metabolism and, consequently, in drug exposure. CYP expression and activities are known to be influenced by non-genetic factors, such as diseases, medication, age, hormones or smoking, leading to phenoconversion and transient poor (or extensive) metabolism despite the wild CYP genotype [14]. Chronic renal disease has been suggested to modify CYP activities through direct inhibition of CYP enzymes and/or by transcriptional down-regulation by uremic toxins and mediators accumulating during renal impairment [15]. Small-molecular weight uremic toxins (indoxyl sulfate, hippuric acid, *p*-cresol, 3-carboxy-4-methyl-5-propyl-2-furanpropanoic acid) alone and particularly in combination decrease the function of CYP1A2, CYP2C9, CYP2E1 and CYP3A4 in hepatic microsomes [16, 17]. Furthermore, human uremic serum has been found to contain mediators (parathyroid hormone, cytokines) that reduce transcriptional CYP expression [18–20]. In rat kidney disease model, reduced binding of nuclear transcription factors, pregnane X receptor (PXR), hepatic nuclear factor 4α (HNF-4α) and RNA polymerase II to CYP2C11 and CYP3A2 promoter as well as diminished histone acetylation was demonstrated, contributing to down-regulation of CYPs [21]. Although ex vivo evidences for uremia-induced modifications of CYP expression appear convincing, we do not have applicable results gathered from human studies. Therefore, attention is necessary when extrapolating from functional data based on the studies with rat hepatocytes and heavily uremic animal models to the clinical care of patients with chronic kidney disease.

Pharmacogenetic approaches identifying loss-of-function (and gain-of-function) alleles of drug-metabolizing enzymes aim to determine permanent poor (and extensive) metabolizer phenotypes; whereas, the expression of these enzymes can provide information about transient modification of metabolizer phenotypes and about phenoconversion of genes more precisely. We have previously described a complex diagnostic tool (CYPtest™) that determines drug-metabolizing capacity by CYP-genotyping for clinically relevant CYP allelic variations and by the current CYP expression in leukocytes. mRNA levels of the major drug-metabolizing CYPs in leukocytes were proven to provide information about the hepatic CYP activities [13]. Continuous refinement of the basic methods for CYP expression is required for reliable estimation of patients’ drug-metabolizing capacity, especially of those whose drug metabolism is expected to be compromised. The aim of the present work was to refine each step of CYP expression measurements (total RNA isolation, reverse transcription and quantitative polymerase chain reaction [qPCR]), and to compare CYP expression (CYP1A2, CYP2C9, CYP2C19 and CYP3A4) in patients with end-stage kidney disease and in organ donors with healthy renal function.

## Materials and methods

### Blood samples

Blood samples from deceased organ donors (*N* = 110) and from patients with end-stage kidney disease (*N* = 105) were obtained from the Department of Transplantation and Surgery, Semmelweis University (Budapest, Hungary). The use of blood samples for scientific research was approved by the Hungarian Committee of Science and Research Ethics. The samples were taken at the time of explantation from hemodynamically stable brain death donors with a normal liver function; whereas, the sampling of the patients with end-stage kidney disease was performed at the time of their admission to the transplantation center for renal transplantation. Demographic data of organ donors and patients were recorded (Table 1). All subjects belonged to the Caucasian white population. The male/female ratio and the average age were similar in the two groups. Leukocytes were isolated from blood samples using red blood cell lysis buffer (Roche Diagnostics, Mannheim, Germany). For optimization of RNA isolation, reverse transcription and quantitative PCR procedures, leukocytes pooled from three healthy volunteers were used.

**Table 1:** Demographic and clinical data of organ donors and patients with end-stage renal disease (ESRD)

	Organ donors	ESRD patients
Number	110	105
Gender: male/female (%)	68/42 (61.8%/38.2%)	57/48 (54.3%/45.7%)
Age at the time of sampling (years)		
Median (min, max)	48 (18, 74)	48 (18, 75)
Cause of death		
Accident, cerebral contusion (%)	28 (25.5%)	
Subarachnoid hemorrhage (%)	25 (22.7%)	
Cerebral hemorrhage (%)	13 (11.8%)	
Subdural hemorrhage (%)	12 (10.9%)	
Stroke (%)	12 (10.9%)	
Aneurysm (%)	9 (8.2%)	
Haemangioma (%)	7 (6.4%)	
Other	4 (3.6%)	
Cause of end-stage renal disease		
Diabetic nephropathy (%)		26 (24.8%)
Hypertensive nephropathy (%)		20 (19.0%)
Polycystic kidney disease (%)		18 (17.1%)
Chronic pyelonephritis (%)		8 (7.6%)
Chronic glomerulonephritis (%)		7 (6.7%)
Nephrotic syndrome (%)		5 (4.8%)
IgA nephropathy (%)		2 (1.9%)
Drug-induced nephropathy (%)		2 (1.9%)
Other (%)		17 (16.2%)

### Total RNA isolation

Six commercially available RNA isolation reagents based on liquid–liquid extraction were compared in the terms of purity, total RNA yield and financial costs. All six reagents contain the combination of phenol with guanidinium thiocyanate: (1) TRIzol™ reagent (Invitrogen/ThermoFisher Scientific, Carlsbad, CA, USA), (2) TRI Reagent^®^ (Molecular Research Center Inc., Cincinnati, OH, USA), (3) Trizolate Reagent^®^ (UD-GenoMed Medical Genomic Technologies Ltd., Debrecen, Hungary), (4) RiboZol^®^ RNA extraction reagent (Amresco LLC/VWR Life Scientific, Solon, OH, USA), (5) RNAzol^®^ RT reagent (Molecular Research Center Inc.) and (6) NucleoZOL reagent **(**MACHEREY–NAGEL GmbH & Co. KG, Düren, Germany). For appropriate comparison of the six RNA isolation reagents, the RNA was isolated from the same amount of cells (10^7^) pooled from three healthy subjects. The leukocytes were suspended in 1 ml of each RNA extraction reagent. RNA isolation was performed according to the manufacturer’s instructions. TRIzol™, TRI Reagent^®^, Trizolate Reagent^®^ and RiboZol^®^ RNA extraction reagent apply chloroform-induced phase separation; whereas, RNAzol^®^ RT and NucleoZOL reagents require RNase-free water for precipitation of DNA and protein contaminants. Further steps of RNA precipitation with 2-propanol, RNA washing with 75% ethanol and RNA reconstitution in RNase-free water were the same in each isolation procedure. RNA content and purity of the samples (*A*_260/280_, *A*_260/230_) were determined based on the UV/Vis spectra of the RNA solutions.

### Reverse transcription

First-strand cDNA synthesis was performed by reverse transcription, and five commercially available cDNA synthesis kits were compared regarding the reverse transcription efficiency and the dynamic range: (1) Maxima First Strand cDNA Synthesis Kit for RT-qPCR (ThermoFisher Scientific, Waltham, MA, USA), (2) qPCRBIO cDNA Synthesis Kit (PCR Biosystems Ltd., London, UK), (3) FastGene Scriptase Basic cDNA Kit (NIPPON Genetics Co. Ltd, Tokyo, Japan), (4) iScript™ cDNA Synthesis Kit (Bio-Rad Laboratories Inc, Hercules, CA, USA) and (5) SensiFAST™ cDNA Synthesis Kit (Bioline GmbH, Luckenwalde, Germany). All five cDNA synthesis kits contain the combination of oligo(dT) with random hexamer primers. To avoid the template variation between assays, the same RNA sample isolated from the leukocyte pool of three volunteers was used for testing the cDNA synthesis kits. Reverse transcription was performed according to the manufacturers’ instruction. A 51.2 pg–4 μg dilution range was assayed, and the dynamic range was determined using six 1:5 serial dilutions of total RNA samples. A standard curve was plotted based on Cq values obtained from qPCR measurements using the diluted and transcribed RNA samples against the log of the total RNA content. The correlation coefficients, the slope of the standard curves and the efficiency were calculated for each cDNA synthesis kit.

### Quantitative real-time PCR assays

For cDNA amplification, the KAPA Probe Fast qPCR kit (Merck KGaA, Darmstadt, Germany) was used. The quantities of CYP mRNAs relative to that of the housekeeping gene glyceraldehyde 3-phosphate dehydrogenase (GAPDH) were determined; therefore, GAPDH expression was set to 1 and CYP mRNA levels were normalized by GAPDH expression. For duplex analysis, the primers and probes for CYP1A2, CYP2C9, CYP2C19 and CYP3A4 were optimized (Table 2). TaqMan probes were labeled with FAM (GAPDH) and HEX fluorophores (CYP probes) (Eurofins Genomics, Ebersberg, Germany). The one-step thermal profile of PCR consisted of 95 °C for 3 min, 50 cycles of 95 °C denaturation for 3 s and 58 °C amplification for 30 s (Bio-Rad CFX96 Touch™ real-time PCR system). The data were analyzed by the Bio-Rad CFX Maestro™ software.

**Table 2:** Sequences of PCR primers and probes for assaying relative CYP mRNA expression

Oligonucleotide	Sequence (5′–3′)
CYP1A2 forward primer	GTC AAT GAC ATC TTT GGA GCA G
CYP1A2 reverse primer	CCT GCC AAT CAC AGT GTC C
CYP1A2 probe	HEX-TGA CAC AGT CAC CAC AGC CAT CTC C-BHQ1
CYP2C9 forward primer	AGA TAG GTA TTA AGG ACA TCA GC
CYP2C9 reverse primer	CCA CTA TGG GTT TCA GGC
CYP2C9 probe	HEX-ACC AAT CTC TCA AAG GTC TAT GGC-BHQ1
CYP2C19 forward primer	ATC AGG ATT GTA AGC ACC C
CYP2C19 reverse primer	TTC TCC AAA ATA TCA CTT TCC AT
CYP2C19 probe	HEX-CCA CTA TCA TTG ATT ATT TCC CGG-BHQ1
CYP3A4 forward primer	TGT CCT ACC ATA AGG GCT T
CYP3A4 reverse primer	CAC AGG CTG TTG ACC A
CYP3A4 probe	HEX-AGT ATG GAA AAG TGT GGG GCT T-BHQ1
GAPDH forward primer	AGC CAC ATC GCT CAG ACA C
GAPDH reverse primer	GCC CAA TAC GAC CAA ATC C
GAPDH probe	FAM-TGG GGA AGG TGA AGG TCG-BHQ1

### Data analysis

CYP expression was determined in 110 organ donors and 105 patients with end-stage renal disease. Statistical analysis was carried out using GraphPad InStat (v3.05; GraphPad Software, San Diego, CA, USA). Distribution of CYP expression data was analyzed by Kolmogorov–Smirnov test, and between-group differences were calculated by Mann–Whitney *U* test. Three categories of CYP expression were applied to describe low, normal and high expressers. The cut-off values for CYP mRNA levels in leukocytes have been previously established on the basis of the cut-off values for the hepatic CYP enzyme activities (CYP1A2: phenacetin *O*-dealkylation; CYP2C9: tolbutamide 4-hydroxylation; CYP2C19: mephenytoin 4′-hydroxylation; CYP3A4: nifedipine oxidation or midazolam 1′- and 4-hydroxylation) [13]. The cut-off values for CYP1A2 (10^–5^ and 5 × 10^–4^), CYP2C9 (2 × 10^–6^ and 10^–5^), CYP2C19 (10^–6^ and 10^–5^) and CYP3A4 (10^–6^ and 10^–4^) in leukocytes allow a distinction between low, normal and high expressers. The prevalence of low, normal and high expressers in patients with end-stage renal disease was compared to that of organ donors by the use of Chi-squared test (GraphPad InStat v3.05, San Diego, CA, USA). A *p* value of < 0.05 was considered to be statistically significant.

## Results

The CYPtest™ diagnostic system has been developed for the estimation of patients’ drug-metabolizing capacity through integrative analysis of clinically relevant mutations in CYP genes and the mRNA expression of major drug-metabolizing CYPs. Peripheral leukocytes are appropriate biological samples for providing information about CYP-mediated drug metabolism because they are easily accessible, display active RNA synthesis and reflect hepatic activities of CYP1A2, CYP2C9, CYP2C19 and CYP3A4 enzymes [13]. Hepatic CYP activities and CYP mRNA concentrations in leukocytes seem to transiently decrease to extremely low levels in patients with some acute or chronic disease. For precise and reliable measurement of low CYP expression in limited amounts of samples, the procedures of RNA isolation, RNA-to-cDNA conversion and qPCR required improvement of the basic CYPtest™ methods.

### Comparison of liquid–liquid RNA extraction methods

The extraction of high-quality RNA is important for a sensitive quantitative analysis of gene expression. Solid-phase extraction kits generally produce high-purity RNA samples; however, liquid–liquid extraction seemed to be more efficient in isolation of transcripts expressed in relatively small amounts. To achieve high-yield and quality of RNA, six commercially available RNA extraction reagents (TRIzol™ reagent, TRI Reagent^®^, Trizolate Reagent^®^, RiboZol^®^ RNA extraction reagent, RNAzol^®^ RT reagent and NucleoZOL reagent) were compared using the same leukocyte pool. RiboZol reagent yielded the highest concentration of total RNA, whereas the RNA yields were significantly lower (*p* < 0.05) using the other reagents (Table 3). The 260/280 and 260/230 ratios are used for the evaluation of the purity of RNA samples, and are expected to be approximately 2 and in the range of 2.0–2.2, respectively. The 260/280 ratios were around 2 except for RiboZol and RNAzol^®^ RT reagents; whereas, the 260/230 ratios were in the acceptable range only in the samples isolated with TRIzol, Trizolate and TRI reagents. Regarding the RNA yield and purity, TRIzol, Trizolate and TRI reagents were considered to be equal; however, TRI reagent was the most favorable in terms of financial cost (Table 3).

**Table 3:** Comparison of RNA extraction kits

RNA extraction reagent	RNA concentration (ng/μl)^a^	260/280 ratio^a^	260/230 ratio^a^	Price of 200 ml reagent (Euro)
TRIzol reagent	259.5 ± 27.43	2.002 ± 0.031	2.007 ± 0.079	580
TRI reagent	235.7 ± 11.94	2.006 ± 0.034	2.115 ± 0.007	182
Trizolate reagent	267.8 ± 22.80	1.992 ± 0.031	2.007 ± 0.051	212
RiboZol reagent	336.2 ± 37.55	1.870 ± 0.101	1.640 ± 0.321	230
RNAzol RT reagent	250.8 ± 41.34	1.795 ± 0.163	1.150 ± 0.330	185
NucleoZOL reagent	256.6 ± 68.98	1.907 ± 0.074	1.413 ± 0.359	230

^a^Mean ± SD of three extractions from the same leukocyte pool

### Optimization of reverse transcription coupled to qPCR

For measuring low CYP expression levels, it is essential to use highly sensitive and efficient reverse transcriptase. Five reverse transcription kits (Maxima First Strand, qPCRBIO, FastGene Scriptase Basic, iScript and SensiFAST cDNA synthesis kits) were tested on the basis of CYP3A4 assay using the RNA sample from the leukocyte pool. The analytical sensitivity, linearity and efficiency of amplification were compared using the 1:5 serial dilutions of RNA ranged from 4 μg to 51.2 pg per reaction. The quality of the standard curves was evaluated using the slope, correlation coefficient (*R*^2^) and the amplification efficiency. The theoretical doubling is expected to produce a standard curve with the slope of − 3.3; whereas, *R*^2^ > 0.9 and the efficiency ranged between 90 and 110% are considered to be acceptable.

**Fig. 1:**
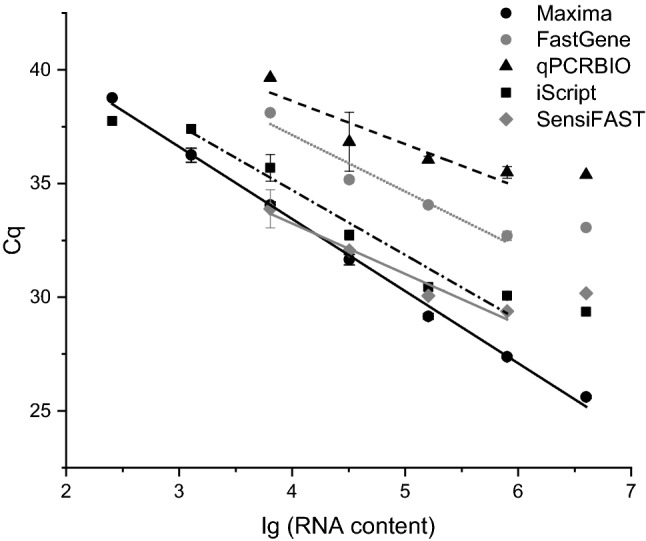
Comparison of reverse transcriptase kits using *C*_q_ (quantification cycle) values for CYP3A4. The 1:5 serial dilutions of the RNA sample were reverse transcribed using Maxima First Strand, qPCRBIO, FastGene Scriptase Basic, iScript and SensiFAST cDNA synthesis kits, and PCR was performed for quantification of CYP3A4. The symbols and whiskers indicate the mean and standard deviation (mean ± SD) of three parallel measurements; whereas, the lines are fitted to the linear concentration range

Transcription of 51.2-pg total RNA per reaction was found to be below the detection limit of CYP3A4 mRNA for all five transcription kits. For qPCRBIO, FastGene Scriptase and SensiFAST cDNA Synthesis kits, the limit of detection was even higher (6400 pg) than for Maxima First Strand and iScript cDNA Synthesis kits (256 pg) (Table 4). Analyzing the linearity of cDNA synthesis, the correlation coefficients (*R*^2^) of the RNA concentration–quantification cycle (Cq) standard curves were higher than 0.9 for Maxima First Strand, iScript and SensiFAST cDNA Synthesis kits; whereas, qPCRBIO and FastGene Scriptase cDNA Synthesis kits displayed weak correlation (Fig. 1; Table 4). The slope of the standard curves and consequently the qPCR efficiency were the closest to the theoretical values (− 3.3 and 100%, respectively) when Maxima First Strand and iScript cDNA Synthesis kits were used for reverse transcription. While qPCRBio, FASTgene Scriptase and SensiFAST cDNA Synthesis kits far exceeded the optimal efficiency. Since the PCR components (primers, probe and PCR reagent) were consistent across all five cDNA synthesis kits, the efficiency differences were attributed to the reverse transcriptase itself. Furthermore, Maxima First Strand cDNA Synthesis kit displayed the widest dynamic range for CYP3A4 expression; therefore, it was used for reverse transcription for the subsequent qPCR measurements.

**Table 4:** Comparison of reverse transcriptase (RT) kits using the standard curves fitted to Cq values for CYP3A4

RT kit	Slope^a^	*R*^*2*a^	Efficiency (%)^a^	Limit of detection (pg of total RNA)	Dynamic range	
					observed	recommended by the manufacturer
Maxima First Strand cDNA Synthesis Kit	− 3.187 ± 0.155	0.996 ± 0.002	106.2 ± 7.08	256	256 pg–4 μg	1 pg–5 μg
qPCRBIO cDNA Synthesis Kit	− 1.648 ± 0.628	0.762 ± 0.192	404.7 ± 251.13	6400	6400 pg–0.8 μg	4 pg–0.1 μg
FastGene Scriptase Basic cDNA Kit	− 1.881 ± 0.307	0.775 ± 0.049	249.3 ± 70.23	6400	6400 pg–0.8 μg	1000 pg–5 μg
iScript™ cDNA Synthesis Kit	− 2.849 ± 0.052	0.958 ± 0.007	124.8 ± 3.35	256	1280 pg–0.8 μg	0.1 pg–1 μg
SensiFAST™ cDNA Synthesis Kit	− 2.218 ± 0.231	0.937 ± 0.037	182.4 ± 32.81	6400	6400 pg–0.8 μg	< 1 μg

^a^Mean ± SD of the dilution set using three RNA samples extracted from the same leukocyte pool

The qPCR methods for CYP expression described by Temesvári et al. [13] applied FAM-labeled probes that did not allow the expression analysis of target and reference genes in a multiplex reaction. In addition to the considerable saving of time, efforts, samples and reagents, a successful multiplex PCR assay provides more precise evaluation of the target gene expression relative to the reference gene. Therefore, the oligonucleotide components of the singleplex qPCR in CYPtest™ were redesigned applying HEX-labeled probes for CYPs and FAM-labeled probe for the reference GAPDH expression (Table 2). The primers were designed in two consecutive exons separated by an intron on the corresponding gDNA (CYP1A2, CYP2C9, CYP2C19) or to the exon–exon junction (CYP3A4, GAPDH); therefore, the amplicon was generated exclusively from the CYP mRNA derived cDNA, and gDNA contamination did not produce false reaction. The redesigned primer–probe combinations and quantification of each gene product were assayed using 5-log serial dilutions of cDNA templates. The amplification efficiency varied between 94.8 and 106.2%, and *R*^2^ was higher than 0.99 for CYP1A2, CYP2C9, CYP2C19, CYP3A4 and GAPDH.

### CYP expression in patients with end-stage kidney disease

**Fig. 2:**
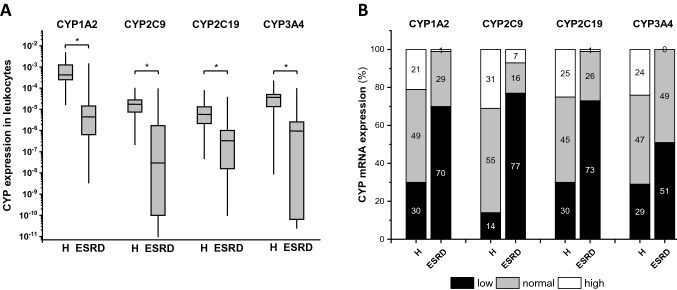
CYP1A2, CYP2C9, CYP2C19 and CYP3A4 expression in leukocytes of the patients with end-stage renal disease (*N* = 105) and of organ donors with normal kidney function (*N* = 110) (**a**), and the ratio of patients/donors expressing CYPs at low, normal or high levels (**b**). **a** CYP mRNA values are presented using the median (line), range (box) and minimum–maximum values (whiskers). Between-group differences were calculated by Mann–Whitney *U* test. **b** The cut-off values for CYP mRNA levels (CYP1A2: 10^–5^ and 5 × 10^–4^, CYP2C9: 2 × 10^–6^ and 10^–5^, CYP2C19: 10^–6^ and 10^–5^, CYP3A4: 10^–6^ and 10^–4^) in leukocytes allowed a distinction between low, normal and high expressers. The numbers indicate the prevalence (%) of low, normal and high expressers in patients with end-stage renal disease and in organ donors. *p < 0.0001; *ESRD* end-stage renal disease; *H* organ donors with normal kidney function

The expression of CYP1A2, CYP2C9, CYP2C19 and CYP3A4 was determined in leukocytes of 105 patients with end-stage renal disease and 110 healthy organ donors (Fig. 2a). The extremely suppressed CYP expression in patients’ leukocytes required method optimization for appropriate detection of CYP mRNAs. The refined methods of RNA extraction using TRI Reagent, reverse transcription using Maxima First Strand cDNA synthesis kit and redesigned oligonucleotide sequences for duplex qPCR (Table 2) were applied for assaying CYP expression. In organ donors, CYP expression displayed approximately a two-magnitude difference between the highest and lowest mRNA levels except for CYP3A4 that exhibited much wider variation (5-magnitude). In patients with end-stage renal disease, high inter-individual variations were observed in CYP expression with 5- to 7-magnitude differences between the highest and lowest levels. Furthermore, in patients with impaired renal function, mRNA levels of all four CYPs were found to be significantly lower than in organ donors. The median values of CYP expression displayed substantial differences between the two groups (*p* < 0.0001) with the greatest in CYP2C9 (600-fold) and the lowest in CYP2C19 (15-fold) (Fig. 2a).

On the basis of CYP expression in leukocytes, hepatic activities of CYP1A2, CYP2C9, CYP2C19 and CYP3A4 were also estimated in patients with end-stage renal disease and in organ donors using the cut-off values for distinction of poor, intermediate and extensive metabolizers described by Temesvári et al. [13]. Comparing organ donors and the patients with end-stage renal disease, significant differences were observed in the proportion of subjects expressed CYP mRNA at low, normal and high levels (Chi^2^ for CYP1A2: 40.2, for CYP2C9: 87.9, for CYP2C19: 47.2, for CYP3A4: 29.9; *df* = 2, *p* < 0.0001) (Fig. 2b). CYP expression was low in most of the patients with impaired renal function (51–77%); while the ratio of high CYP expressers was 0–7%. In organ donors with healthy renal function, we found much less subjects with low CYP expression (30% or less), and the number of high expresser subjects was also remarkable (21–31%).

## Discussion

For personalized medication, it is essential to obtain information about patients’ drug-metabolizing capacity [14, 22]. Hepatic enzymes belonging to CYP1–3 families are primarily responsible for the biotransformation of drugs with major contributions of CYP3A4/5, CYP2C9, CYP2C19, CYP2D6, CYP1A2 and CYP2B6; therefore, any information on hepatic CYP activities is of significant importance for the estimation of drug-metabolizing capacity [12]. The first step for the estimation of CYP status and drug-metabolizing capacity is the identification of clinically relevant CYP alleles resulting in reduced and no enzyme activities or even extensive and ultrafast metabolism [23, 24]. Transient decrease (or elevation) of hepatic CYP activities as a consequence of non-genetic factors can partly be assessed from CYP expression in leukocytes. Although CYP expression and activities in leukocytes are by magnitude much lower than in the liver, the leukocyte expression of several CYPs (CYP1A2, CYP2C9, CYP2C19 and CYP3A4) appears to reflect the hepatic activities [13]. It should be noted that leukocytes are inappropriate cells for the assessment of hepatic CYP2B6 and CYP2D6 activities because there are no association between their hepatic activities and leukocyte expression [13]. Furthermore, patients’ CYP3A-substrate-metabolizing capacity depends on the activity of CYP3A4 rather than CYP3A5 because (1) the amount of CYP3A4 in hepatic CYP3A pool substantially exceeds that of CYP3A5 (85 and 5%, respectively) [25]; (2) due to the defective CYP3A5*3 allele common in Caucasian white populations (88–97%) [12], CYP3A5 expression is restricted to those subjects who carry the functional wild-type CYP3A5*1 allele; (3) the activity of CYP3A5 is substrate specific. Functional CYP3A5 (CYP3A5*1 allele) does not influence the pharmacokinetics or pharmacological effects of several CYP3A substrates (clonazepam, fluvastatin, pravastatin, carbamazepine), whereas the clearance of others, such as tacrolimus, by CYP3A5 is significantly higher than by CYP3A4; therefore, CYP3A5*1 carrier patients require double dose of tacrolimus for therapeutic blood concentration than those with CYP3A5*3/*3 genotype. It also means that identification of CYP3A5*1 is appropriate for the estimation of tacrolimus-metabolizing capacity, and no further CYP3A5 expression analysis is required [26].

Detection of permanent (genetic) variations and transient changes in CYP expression can facilitate the identification of high-risk patients [26–29]. Due to the potential accumulation of uremic toxins, CYP genes were expected to exhibit low expression in leukocytes of patients with impaired kidney function. Therefore, the workflow steps of CYP expression analysis using the qPCR technique (RNA extraction, reverse transcription and qPCR for various CYPs) required refinement of the method described by Temesvári et al. [13]. For gene expression analysis, separation of RNA from other cellular macromolecules (DNA, proteins) by either liquid–liquid or solid-phase extraction are critical for yielding high-quality RNA in sufficient amount. Various solid-phase extraction methods are available that can isolate high-purity RNA samples. Besides further advantages of solid-phase extraction, such as good reproducibility, reduction of hazardous chemical consumption and easy automation, the low yield of RNA is a major drawback [30, 31]. By solid-phase method, there is often no chance for extraction of target RNA expressed at low concentration. Liquid–liquid extraction generally yields significantly higher amount of RNA than solid-phase extraction methods [32]; however, to obtain high-purity RNA samples, a judicious choice of the most suitable liquid–liquid extraction method is required. The most common methods apply phenol–guanidinium thiocyanate reagents with or without phase-separation by the addition of chloroform. Although the RNA extraction kits tested in the present study followed the same subsequent steps of RNA precipitation from the aqueous phase with 2-propanol and RNA purification with ethanol [33–35], the RNA yield and purity from leukocytes were different. RiboZol reagent yielded the highest quantity of RNA; whereas, TRIzol, Trizolate and TRI reagents produced RNA with the lowest contamination. TRI reagent was nevertheless the most advantageous because of the reasonable financial cost. gDNA impurities in RNA samples can significantly impact the qPCR leading to overestimation of target mRNA expression and to inaccurate expression results. Contaminating gDNA can be estimated by PCR assays omitting reverse transcription in parallel of qPCR coupled with reverse transcription; however, the additional assay is costly and consumes RNA sample available in limited amount. An alternative method is the RNase-free DNase digestion of RNA samples prior to reverse transcription and qPCR assay. Although the DNase treatment followed by DNase inactivation can substantially reduce gDNA contamination, the additional steps increase the time of sample preparation procedure. gDNA insensitive assay with proper design of primers is a time and reagent saving alternative method that is applicable for many eukaryotic genes [36]. Designing a primer sequence at exon–exon junction or including a large intron between the forward and reverse primers can ensure the amplification exclusively from cDNA; therefore, gDNA does not interfere with gene expression analysis. Since CYPs and the reference GAPDH are multi-exon genes, we could design gDNA insensitive assays.

Reverse transcription of RNA template is another crucial step in quantitative assay of gene expression. The reverse transcriptase enzyme is generally expected to be efficient in a wide dynamic range converting high- and low-abundance transcripts into cDNA [37–39]; however, the manufacturers’ specifications inform merely about the total RNA quantity and not about the specific target transcript that is capable to be reverse transcribed by the particular enzyme. Furthermore, the manufacturers’ specifications rarely provide information whether the reverse transcription product is directly adaptable to qPCR or post-reverse transcription processing (dilution or cleaning) is required [38]. For CYP expression analysis, the major suitability criteria for reverse transcriptase were the enzyme efficiency with wide dynamic range and direct PCR-adaptability. All five cDNA synthesis kits tested in the present study contained modified MMLV (Moloney Murine Leukemia Virus) reverse transcriptase which has been reported to display higher cDNA synthesis rate than that of AMV (Avian Myeloblastosis Virus) [40, 41]. Furthermore, all cDNA synthesis kits apply the same priming strategy with the combination of random hexamer and anchored oligo(dT) primers that can provide optimal sensitivity and accuracy of first strand cDNA synthesis. Despite the same type of reverse transcriptase and priming, these cDNA synthesis kits showed considerable variations in efficiency, dynamic range and detection limit. iScript™ and Maxima First Strand cDNA Synthesis kits displayed the lowest detection limit; however, the efficiency and the dynamic range of iScript™ lagged behind those of Maxima First Strand cDNA Synthesis kit. Maxima First Strand cDNA Synthesis kit appeared to be the most efficient with the widest range for quantification of target transcript. A major difference between cDNA synthesis kits is the RNase H activity degrading the original RNA template after cDNA synthesis. iScript™ and Maxima First Strand cDNA Synthesis kits retained the RNase H activity; whereas, reverse transcriptase in qPCRBIO, SensiFAST™ cDNA Synthesis kits and FastGene Scriptase Basic cDNA kit has reduced RNase H activity. Reduced or no RNase H activity is an advantage in production of large cDNA; however, it entails a disadvantage to the subsequent quantitative PCR, because the RNA template can bind to the cDNA and blocks the primer binding to the cDNA template during PCR [36]. An additional cleaning step is required before qPCR that increases the cDNA production time and the waste of cDNA. Therefore, a first-strand cDNA synthesis kit having RNase H activity is an appropriate choice of reverse transcription directly coupled with qPCR. The background RNA content in reverse transcription can also influence the sensitivity of subsequent PCR [37]; however, CYP expression was determined relative to the reference GAPDH expression in duplex assays, and the amount of background RNA was, thus, the same for the expression analysis of the target and the reference genes.

To obtain reliable CYP expression results even in a broad range of transcript amounts, it was essential to systematically optimize RNA extraction and reverse transcription coupled to qPCR. Careful application of TRI Reagent yielded sufficient amount of high-quality RNA from leukocytes, and reverse transcription by Maxima First Strand cDNA Synthesis kit efficiently produced cDNA template directly transposable to qPCR; whereas, the detection of various CYPs and the reference GAPDH in duplex PCR improved the assay reliability. The refined CYP expression analysis indicated a wide concentration range of CYP mRNAs in leukocytes of patients with impaired kidney function. Furthermore, the average CYP expression was significantly lower in patients with end-stage kidney disease than in organ donors. Our findings are considered to be a direct evidence of transcriptional down-regulation of *CYP1A2*, *CYP2C9*, *CYP2C19* and *CYP3A4* genes in patients with kidney dysfunction, confirming some of the clinical observations of reduced CYP-mediated drug metabolism [11, 42–47]. As a consequence of a decrease in CYP2C9 activity, the clearance of the anticoagulant *S*-warfarin was reported to be substantially reduced in patients with end-stage renal disease [43, 48], and the warfarin dose requirements of patients with moderate and severe kidney impairment were significantly lower than those of no kidney disease [43]. However, the impact of renal disease induced CYP2C9 down-regulation on the clearance of the anti-hyperglycemic tolbutamide is not clear in human, because tolbutamide is almost exclusively eliminated by the kidneys, and is, therefore, contraindicated in patients with severe renal failure [49]. In rats, CYP2C11 down-regulation was also demonstrated in chronic renal disease; however, no change in hepatic tolbutamide 4-hydroxylation was observed most probably due to the fact that tolbutamide is not selective for CYP2C11 contrary to the human CYP2C9 ortholog, but other CYPs are involved in the metabolism in rat [50]. Furthermore, chronic kidney disease resulted in substantial reduction of CYP3A2 expression and activities (erythromycin *N*-demethylation; 1′- and 4-hydroxylation of midazolam) in rats [50, 51]. In patients with end-stage renal disease, erythromycin *N*-demethylation by CYP3A4 was also reported to decrease; however, the alteration of erythromycin clearance was not exclusively attributed to the reduced CYP3A4 activity, but also to the changes in transporter activities (OATPs, P-gp) [44]. Increased systemic exposure of several other CYP3A4-substrate drugs, such as tadalafil and solifenacin, was demonstrated in patients with chronic renal impairment [52]. In contrast, the clearance of the CYP3A4 probe substrate midazolam appeared to be unchanged in kidney disease patients [53]. Regarding hepatic CYP1A2 expression, complex impact of chronic kidney disease was depicted: (1) human uremic serum significantly decreased the expression and the activity of CYP1A2 in rat hepatocytes [18]; (2) in rats, mild or negligible effect of renal impairment on CYP1A2 expression was observed in vivo [21, 51]; (3) in patients with chronic kidney disease, mild or no changes were observed in clearance of CYP1A2 substrates, such as duloxetine, lidocaine or tasimelteon [54].

In the present study, the prevalence of patients with low CYP expression was found to be considerably higher in the end-stage kidney disease group than in organ donors. Approximately, one quarters of organ donors expressed CYPs at high concentration; whereas, high CYP expression was scarcely identified in patients with impaired kidney function. As a consequence of reduced drug-metabolizing capacity, altered drug exposure, hence increased risk of overdosing may occur; therefore, the dosing regimen is recommended to be adjusted in patients with chronic kidney disease to avoid adverse drug reactions [47, 55]. The non-renal clearance of drugs, such as duloxetine, erythromycin, ciprofloxacin or warfarin, has been reported to decrease, and reduced dosing is required in patients with severe chronic kidney disease [43–46]. It should be noted that renal replacement therapy can complicate the medication of patients. Kidney transplantation can substantially and stably ameliorate CYP expression and drug-metabolizing capacity; whereas, intermittent dialysis can transiently increase CYP-mediated drug metabolism; however, the duration and the extent of the alteration can be poorly quantified [10, 55].

Some limitations of the clinical part of the present work should be mentioned. First, for comparison of CYP expression in patients with end-stage renal disease, deceased organ donors and not healthy volunteers were enrolled in the healthy control group. However, all the donors were considered to be healthy on the basis of the normal liver and kidney function parameters. For better comparison, further study involving healthy subjects may be required. Second, although direct evidence has previously been provided for the correlation between leukocyte expression and hepatic enzyme activities (as well as hepatic expression) of CYP1A2, CYP2C9, CYP2C19 and CYP3A4 [13], it should be validated for patients with end-stage renal disease. Taken liver biopsy sample for CYP expression assays is risky and definitely not allowed to obtain from patients because of ethical issues. Further studies are nevertheless required to provide indirect evidence by obtaining significant correlation between the leukocyte CYP expression in patients and the elimination rate of CYP-selective substrate drugs.

In sum, qPCR technique is considered to be a sensitive and accurate method for gene expression analysis. The refinement of RNA extraction and reverse transcription as well as the development of duplex qPCR assays substantially improved the detection of low-level CYP expression even with low input RNA amounts. Poor CYP expression was expected in leukocytes of patients with end-stage renal disease; however, the expression of CYP1A2, CYP2C9, CYP2C19 and CYP3A4 was efficiently quantified using the refined method. The expression of these CYP species was significantly lower in patients with end-stage renal disease than in organ donors with normal kidney function. Our findings provided a direct evidence for transcriptional down-regulation of CYP genes in patients with impaired kidney function that can consequently contribute to the increase of drug exposure. Information on patients’ CYP expression in combination with identification of polymorphic CYP alleles may refine the personalized medication, facilitating the appropriate dosage, and can predict the risk of outlying from the therapeutic concentration range.

## Abbreviations

Cq: Quantification cycle
CYP: Cytochrome P450
GAPDH: Glyceraldehyde 3-phosphate dehydrogenase
gDNA: Genomic DNA
PCR: Polymerase chain reaction
qPCR: Quantitative PCR
